# Light Quality Effect on Internal N Retranslocation in *Podocarpus macrophyllus* Precultured with Exponential Nutrient Loading

**DOI:** 10.3390/plants13050705

**Published:** 2024-03-01

**Authors:** Yige Wang, Xiangyang Sun, Suyan Li

**Affiliations:** Key Laboratory for Silviculture and Conservation of Ministry of Education, College of Forestry, Beijing Forestry University, Beijing 100083, China; amanoyuk1teru@bjfu.edu.cn

**Keywords:** exponential fertilization, ornamental plant, root allocation, urban greening, light spectra, shoot to root ratio

## Abstract

Streetlamp light is inevitable in the night landscape of a city and may affect the phenology of newly planted ornamental plants, but it has rarely been fully examined. Newly transplanted ornamental plants probably suffer periodic shocks, which mainly result from the inefficient reuse of internal nutrients for new growth. Exponential nutrient loading (ENL) is well known for its ability to overcome transplant shocks by promoting retranslocation for the reuse of strengthened nutrients from internal reserves in precultured seedlings. Transplantation to urbanized lands is distinct from that of montane areas; this is mainly due to a high frequency of exposure to the artificial illumination of night lighting. It is suspected that this lighting modifies vegetative phenology and generates potential risks by increasing reliance on internal nutrient retranslocation. In this study, *Podocarpus macrophyllus* seedlings were cultured with ENL at low and high rates of nitrogen (N) deliveries (40 and 120 mg N seedling^−1^, respectively), and the high-rate treatment was identified as being able to trap seedlings within toxic states. A labeled ^15^N isotope was pulsed to transplanted seedlings exposed to simulated light qualities in red, green, and blue light spectra. The seedlings harvested at one month showed rare responses to the interactive spectra and preculture treatments, but most of them responded to the low-rate N preculture treatment with stronger abilities in terms of the reuse of internal N and the synthesizing of photosynthetic pigments. In conclusion, it was verified that night light enforces the effect on newly transplanted plants; the red light invoked internal N for reuse, and the blue light promoted the uptake of the current N. The internal N reserve established through preculture ENL rarely made a contribution to the night light effect, except for the enhancement of height growth in the red light. The red light spectrum was recommended for the exposure of newly transplanted seedlings due to its effect on the enhancement of the retranslocation of internal N and the induction of a steady state of uptake from the current N input.

## 1. Introduction

Urbanization is the driver of social evolution and economic development. It has also been given a special meaning in the recovery from the pessimistic attitude that has developed as a consequence of the post-pandemic low economic performance. Greening is a direct practice that constructs green spaces in newly built areas in the urbanizing regions of a sprawling city. Natural regeneration is being employed in the remnant forests of many cities of the world, but it has limits in terms of species control and usually results in domination by more invasive species [1]. In contrast, tree planting is a more flexible approach that can replenish species and shape urban forest composition with more desired species [1,2,3]. The first challenge that newly planted trees may face is the shortage of available nutrients in the soils of urbanized lands [4,5]. This was partly determined by studying the high heterogeneity in the distribution pattern of soil nutrient availabilities [4]. Another possible reason for the shortage is that it is a result of the unordered forms of necessary nutrients in soils that have been subjected to intensive anthropogenic activities [5]. In addition, strongly growing weeds generate a competition not only for aerial light resources but also for the soil nutrients that must be acquired [6,7]. Streetlamp light is an inevitable factor that newly planted plants need to face; it functions as a supplemental light and thus prolongs the photoperiod and increases the demand for the nutrients needed to fuel accelerated carbon (C) assimilation [8,9,10]. The current evidence is insufficient and not able to identify whether night light promotes acclimation or whether it enhances low nutrient stress. It is necessary to examine the post-transplantation responses of newly planted trees following the shortage in nutrient supply experienced by juvenile plants dwelling in city soils. 

Nutrient loading is a practical practice in which the seedling culture is fed with more nutrients than are needed via a large number of applications in order to establish a reserve that can be used to overcome transplant shock [11,12]. This approach was developed in contrast to the conventional fertilizer regime, which only delivered nutrients several times but with a large amount in each application; this practice easily resulted in fertilizer waste through leaching and a low uptake efficiency [13,14,15]. As plants accumulate biomass in a reserve at exponentially increasing rates, the nutrient loading was modified so that it could be employed at an exponential rate; this is known as exponential nutrient loading (ENL) or exponential fertilization [16,17]. The abundant storage of nutrients established through ENL has been identified as being capable of successfully fueling the early growth of newly planted seedlings and strengthening them with a higher ability to cope with abiotic stresses [18,19,20]. However, the ability of a seedling to reserve nutrients does not always correspond with the delivery rate through ENL. Changes in dry mass accumulation and nutritional state along the increasing gradient of supply can be synthesized into independent states. Empirical studies have summarized these states as the stages of deficient uptake, luxury consumption, and overdose toxicity [21,22,23]. The optimum rate of supply was characterized as the critical value before toxicity, beyond which any higher rate of supply would result in a deficit of dry mass accumulation and poor transplant performance [11,14,16,20]. Most of the abovementioned findings were obtained from studies involving tree planting programs for restoration in montane areas, reforestation on abandoned lands, and the establishment of plantations. To our knowledge, this has rarely been tested in urban greening trials and even less is known about whether ENL can also be effective in the promotion of transplantation performance under the conditions of exposure to artificial illuminators. 

At the early post-transplantation stage, seedlings need to remobilize their inner stored nutrients to fuel new growth in order to assimilate the current resources [16]. In this period, the new roots that have just commenced their initial growth and the root system are not at a level which is mature enough to support nutrient foraging in complicated soil environments [13,24]. Therefore, seedlings precultured with ENL usually obtain a higher ability for nutrient retranslocation because of more abundant and enhanced internal reserves [25,26]. It is widely agreed that retranslocation is enhanced with time due to the increasing demand for new growth; hence, sink strength is the driver of internal nutrient cycling [27,28]. From this perspective, exposure to a prolonged photoperiod can be understood as a promotion of sink strength through the acceleration of shoot elongation [12,29,30]. More needs to be known about the effect of the night light spectrum on newly planted ornamental seedlings precultured with ENL. 

Night light is an indicator of the urbanization and economic state of a given city [31,32]. Streetlamps that illuminate with light of medium to high correlated color temperatures were identified as being attractive for passersby and better able to meet their psychological preferences [33]. Hence, the city dwellers’ experiences of night light are important for their perceptions of their quality of life and their subjective well-being [34,35]. However, it was also argued that streetlamp light was a source of light pollution for the night landscape [36,37]. This was partly identified because night light impaired the sustainable development of the habitats and community activities of urban animals, such as bats [38], birds [39], flies [40], crickets [41], etc. The impact of night light can be as large as it is remarkable in urban ecosystems that are supported by green spaces with greeneries [42]. Exposure to light sources at night is an artificial intervention into the ordinary growing rhythms of the phenology and development of street trees [8]. It has been confirmed that the direct exposure to the artificial light provided by streetlamps interrupted the autumn phenology and the length of the vegetative season [43]. However, it appears that this interruptive effect was species-specific [44]. The relevant issues have not attracted very much attention, but for practical and sustainable reasons, it is necessary to determine the mechanisms that account for the night light effects on tree physiology. A specific spectrum in an extended photoperiod can further activate hydraulic cycling, biomass accumulation, and internal nitrogen retranslocation [8,9]. Therefore, these factors, when considered together, suggest that seedlings newly transplanted to a stand exposed to streetlamps can probably experience an inevitable force that strengthens sink demand and, subsequently, the internal nutrient retranslocation. The extent to which night light affects preculture with ENL is still unknown. It is also unclear what the nutrient recovery rate is during the process from preculture to post-transplantation. Confirmative findings need to be verified using seedlings precultured with ENL; such a verification, however, is rarely documented in the current literature. 

The abovementioned issues included species-specific tests on nutrient establishment through ENL during preculture and post-transplantation exposure to an extended photoperiod; both of these needs were identified using ornamental species in a simulated post-transplantation experiment. In this study, *Podocarpus macrophyllus* was chosen as a typical ornamental plant species as it had been proven that it showed significant responses to precultured ENL [13,23,29] and a remarkable ability to cope with transplant shocks [4,24,45]. Nitrogen (N) was focused on as a typical macro-nutrient that is greatly needed by tree seedlings from nursery culture to the post-transplantation stage [11,22,27]. A simulation experiment was conducted in a highly controlled environment to raise seedlings with ENL and to transplant them so that they could receive varied light spectra. The transplanted seedlings were harvested twice to mimic the two critical periods in which the sink demand may have been changed. Our objectives were to examine the effects of night light spectra on the internal N retranslocation according to the physiological responses in the transplanted seedlings precultured with ENL.

## 2. Results

### 2.1. Nutritional Characteristics in Precultured Seedlings

**Figure 1:**
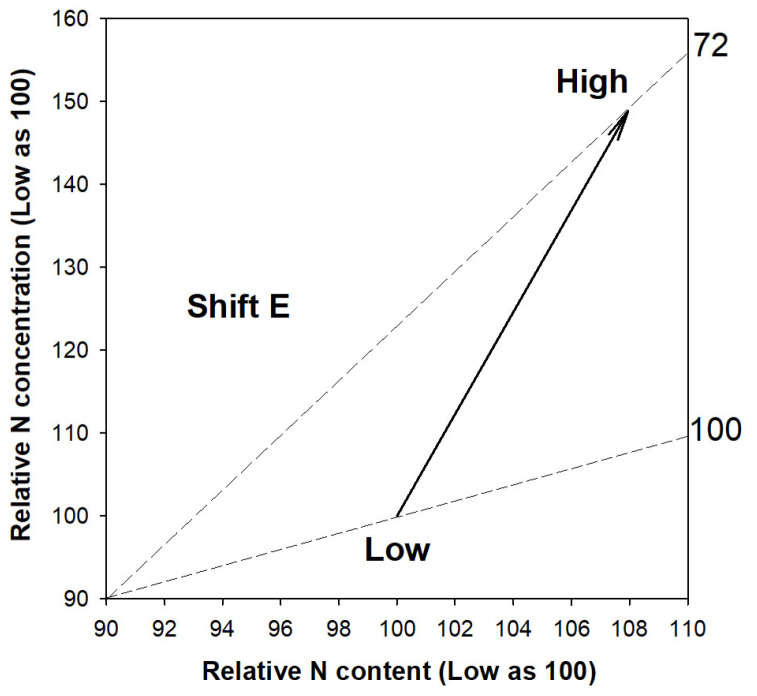
Vector analysis of synthesized relative N content (x-axis), N concentration (y-axis), and biomass (end of dashed line) in shoots of *Podocarpus macrophyllus* seedlings subjected to ENL at high-rate N application (120 mg N seedling^−1^) relative to those at low rate (40 mg N seedling^−1^). Nomograph vectors indicate a relative nutritional state of toxic accumulation (Shift E) diagnosed possibly by relative excess N supply [22].

Compared to the high-rate N application (individually 120 mg N) through ENL, the low rate of N delivery (individually 40 mg N) led to higher shoot height growth, diameter of the root collar (RCD), and biomass of the shoot (Table 1). In contrast, the low rate of N delivery reduced the N concentration in both the shoot and root parts. The synthesized changes in biomass, N content, and N concentration can be characterized as a relative overdose of N supply, leading to an excessive level that causes a relative toxic accumulation of N in the seedlings subjected to a high-rate N application compared with a low rate (Figure 1). Therefore, it is identified that the high rate used in this study provides N at an overuse rate that is relative to the low rate of N application. 

### 2.2. Height, RCD, and Biomass in Transplant Seedlings

**Figure 2:**
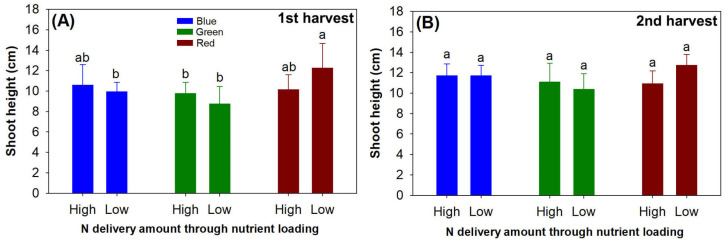
Shoot height in transplanted *Podocarpus macrophyllus* seedlings precultured with ENL at high (120 mg N seedling^−1^) and low rates (40 mg N seedling^−1^) of N application and subjected to different light-emitting diode (LED) spectra in red, green, and blue colors in both harvests (1st harvest, (**A**); 2nd harvest, (**B**)). Error bars present standard errors, above which are lowercase letters indicating significant difference identified using Tukey test at 0.05 level.

The current light-emitting diode (LED) spectra and the N rate of ENL in the preculture generated an interactive effect on shoot height in the first harvest of the post-transplantation seedlings (*F* = 4.87; *p* = 0.0106) (Figure 2A). The precultured seedlings with a low N rate that were exposed to red light spectrum showed greater shoot height than those exposed to a green light spectrum and those precultured with a low rate of N in blue light. However, in the second harvest, no difference was detected in terms of the effects of either the current light spectra (*F* = 1.27; *p* = 0.2866) or the preculture N rate of ENL (*F* = 0.38; *p* = 0.5389) (Figure 2B). 

**Figure 3:**
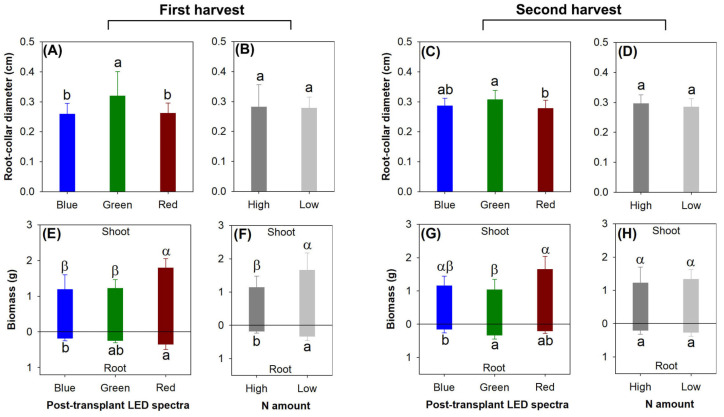
Reponses of root collar diameter (RCD) (**A**–**D**) and biomass (**E**–**H**) in *Podocarpus macrophyllus* seedlings to current light spectra (**A**,**C**,**E**,**G**) and preculture rates of N delivery through ENL (**B**,**D**,**F**,**H**) in both harvests (1st harvest, (**A**); 2nd harvest, (**B**)). Error bars present standard errors, above which are different letters indicating significant difference identified using Tukey test at 0.05 level. Different shoot biomasses were marked by different Greek letters, and different root biomasses were marked by different lowercase letters.

The current light spectra exposed the significant effects on RCD in both harvests (first, *F* = 6.78, *p* = 0.0021; second, *F* = 5.25, *p* = 0.0077), but the preculture rate of N delivery did not modify RCD (first, *F* = 0.04, *p* = 0.8425; second, *F* = 1.94, *p* = 0.1684) (Figure 3A–D). Among the three LED light spectra, RCD was the highest in the green light spectrum in the first harvest, but RCD was higher in the green light spectrum than in the red light spectrum only in the second harvest (Figure 3A,C). 

In the first harvest, either the current light spectra or the preculture N delivery rate generated the main effects on the biomass in the shoot (Light, *F* = 4.05, *p* = 0.0453; N, *F* = 7.22, *p* = 0.0198) and root parts (Light, *F* = 7.48, *p* = 0.0078; N, *F* = 15.64, *p* = 0.0019) (Figure 3E,F). Among the three types of LED light spectra, the red light spectrum led to greater biomass relative to the blue light spectrum in both the shoot and root parts, and the shoot biomass was also greater in the red light spectrum compared with that in the green light spectrum (Figure 3E). The low rate of N delivery led to greater biomass in both the aboveground and belowground parts relative to the high-rate application (Figure 3F).

In the second harvest, the current light spectra still generated the main effects on the biomass in the shoot (*F* = 4.33, *p* = 0.0384) and root parts (*F* = 4.14, *p* = 0.0429) (Figure 3G), but the preculture N delivery rate lost its effect on the biomass (shoot, *F* = 0.42, *p* = 0.5315; root, *F* = 1.08, *p* = 0.3188) (Figure 3H). In the shoot parts, the red light spectrum led to greater biomass compared with the green light spectrum in the aboveground parts; in the roots, the biomass was also greater in the red light spectrum than in the green light spectrum (Figure 3G). 

### 2.3. Nitrogen Content and Concentration in Transplant Seedlings

**Figure 4:**
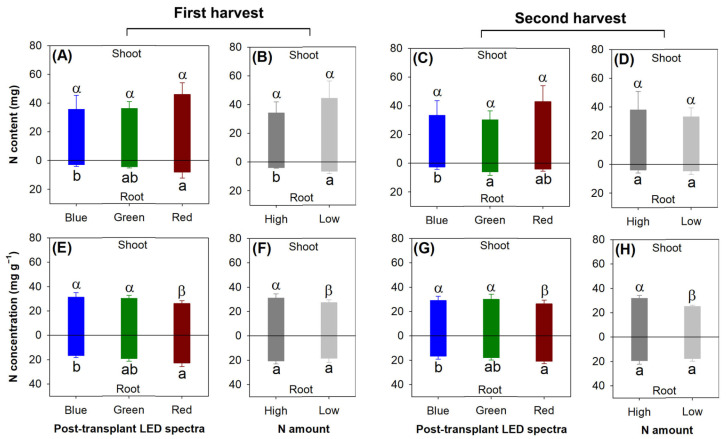
Nitrogen content (**A**–**D**) and N concentration (**E**–**H**) in *Podocarpus macrophyllus* seedlings exposed to current light spectra (**A**,**C**,**E**,**G**) and preculture rates of N delivery through ENL (**B**,**D**,**F**,**H**) in both harvests (1st harvest, (**A**); 2nd harvest, (**B**)). Error bars present standard errors, above which are different letters indicating significant difference identified using Tukey test at 0.05 level. Shoots are marked by different Greek letters, and roots are marked by different lowercase letters.

The shoot N content was not affected by either the current light spectra (first, *F* = 1.68, *p* = 0.2280; second, *F* = 2.42, *p* = 0.1309) or the preculture N application rate in both harvests (first, *F* = 3.93, *p* = 0.0707; second, *F* = 0.96, *p* = 0.3461) (Figure 4A–D). However, the light spectra modified the N content in the roots, which was increased in the red light spectrum compared with that in the roots exposed to the blue light spectrum in the first harvest (*F* = 7.48, *p* = 0.0078). The green light spectrum also led to higher root N content compared with the blue light spectrum in the second harvest (*F* = 4.31, *p* = 0.0389) (Figure 4A,C). In addition, the root N content was also higher in the low-rate delivery of N through ENL than in the high-rate treatment in the first harvest (*F* = 5.23, *p* = 0.0412) (Figure 4B). 

The red light spectrum led to lower N concentration in the shoots compared with that in the blue and green light spectra in both harvests (first, *F* = 7.40, *p* = 0.0081; second, *F* = 8.52, *p* = 0.0050) (Figure 4E,G). The red light spectrum also led to a higher N concentration in the roots compared with that in the blue light spectrum in both harvests (first, *F* = 9.69, *p* = 0.0031; second, *F* = 5.79, *p* = 0.0174). The concentration of N in the shoots was increased by the high rate of N delivery through ENL relative to the low rate of N application in both harvests (first, *F* = 10.83, *p* = 0.0064; second, *F* = 91.54, *p* < 0.0001) (Figure 4F,H). 

### 2.4. Nutritional Characteristics in Transplanted Seedlings

**Figure 5:**
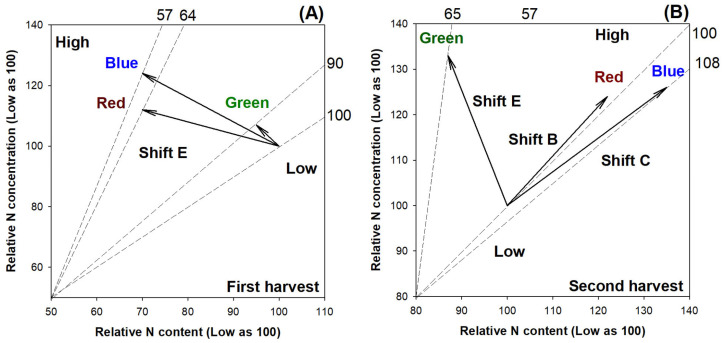
Vector analyses of synthesized relative N content (x-axis), N concentration (y-axis), and biomass (end of dashed line) in shoots of *Podocarpus macrophyllus* seedlings subjected to current light spectra and ENL at high rate of N application (120 mg N seedling^−1^) relative to those at low rate (40 mg N seedling^−1^) in first (**A**) and second harvests (**B**). Nomograph vectors indicate relative nutritional states. Interpretations: Shift B, steady-state uptake of N to a sufficient level; Shift C, requirement of N is limited due to the deficiency; Shift E, toxic accumulation of N caused by excess supply [22].

In the first harvest, all three types of light spectra reduced the N content and biomass but increased the N concentration in the low-rate application of N relative to the high-rate N delivery (Figure 5A). These were characterized as symptoms of the overdose supply of N, which exceeded demand and caused toxicity. In the second harvest, however, the relative nutritional states varied in response to the different light spectra (Figure 5B). Relative to the low rate of N delivery, the high-rate application in the green light spectrum resulted in symptoms caused by the excessive supply (Shift E), but the red light spectrum induced a steady-state delivery of N (Shift B), and the blue light spectrum resulted in nutrient deficiency (Shift C).

### 2.5. Impacts of Spectrum and Preculture N Delivery on N Retranslocation

**Figure 6:**
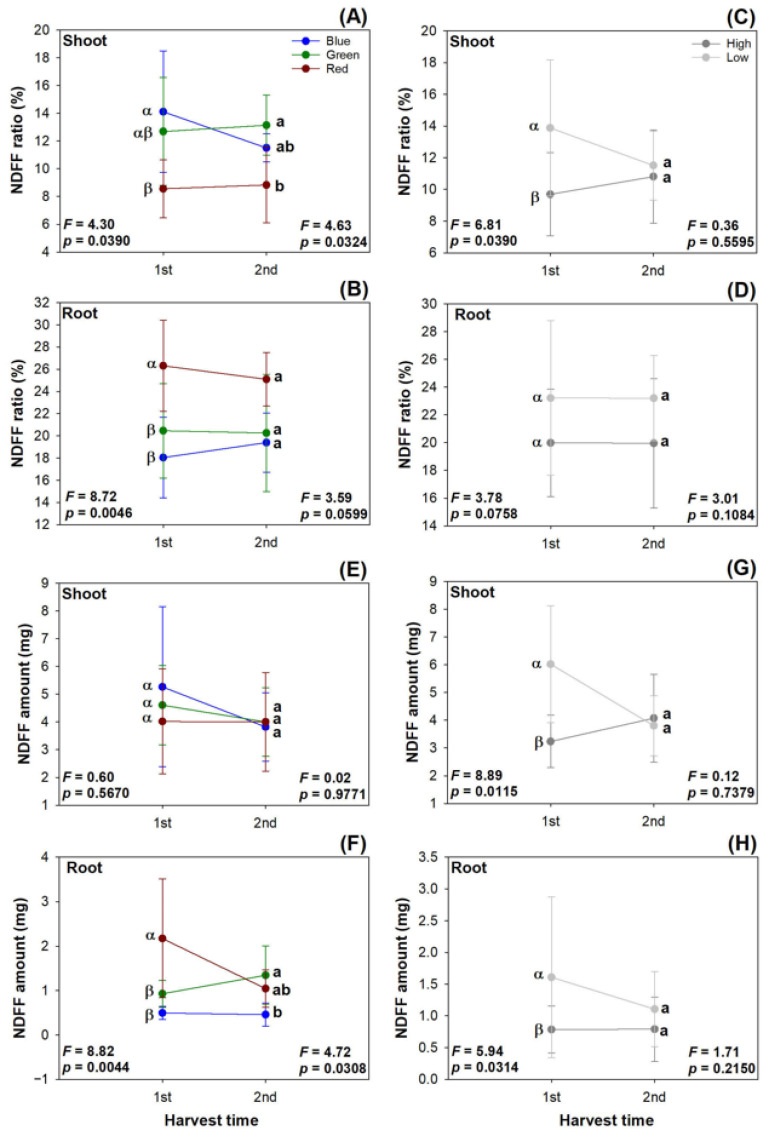
Dynamic changes in nitrogen derived from fertilizer (NDFF) ratio (**A**–**D**) and amount (**E**–**H**) in transplanted *Podocarpus macrophyllus* seedlings subjected to current light spectra (**A**,**B**,**E**,**F**) and preculture N delivery through ENL at high and low rates (**C**,**D**,**G**,**H**) in both harvests. Error bars present standard errors, above which are different letters indicating significant difference identified using Tukey test at 0.05 level. Data from first harvest are marked by different Greek letters, and those from second harvest are marked by different lowercase letters.

The nitrogen derived from the fertilizer (NDFF) ratio in the shoots was lowered in the red light spectrum compared with that in the blue light spectrum in the first harvest, but the red light spectrum led to a lower shoot NDFF ratio compared with that in the green light spectrum in the second harvest (Figure 6A). In contrast, the red light spectrum elevated the NDFF ratio in the roots relative to that in the other two spectra in both harvests (Figure 6B). The low rate of the preculture N application resulted in a higher NDFF ratio in the shoots in the first harvest compared with the high rate of N delivery through ENL (Figure 6C). 

The current light spectra did not affect the shoot NDFF amount in the two harvests (Figure 6E). The root NDFF amount was increased in the red light spectrum relative to that in the green and blue light spectra in the first harvest, but the root NDFF amount was increased by the green light spectrum compared with that in the blue light spectrum in the second harvest (Figure 6F). The low rate of N delivery increased the NDFF amount in the shoots and roots compared with the high rate of N application in the first harvest, but this effect was diminished in the second harvest (Figure 6G,H). 

**Figure 7:**
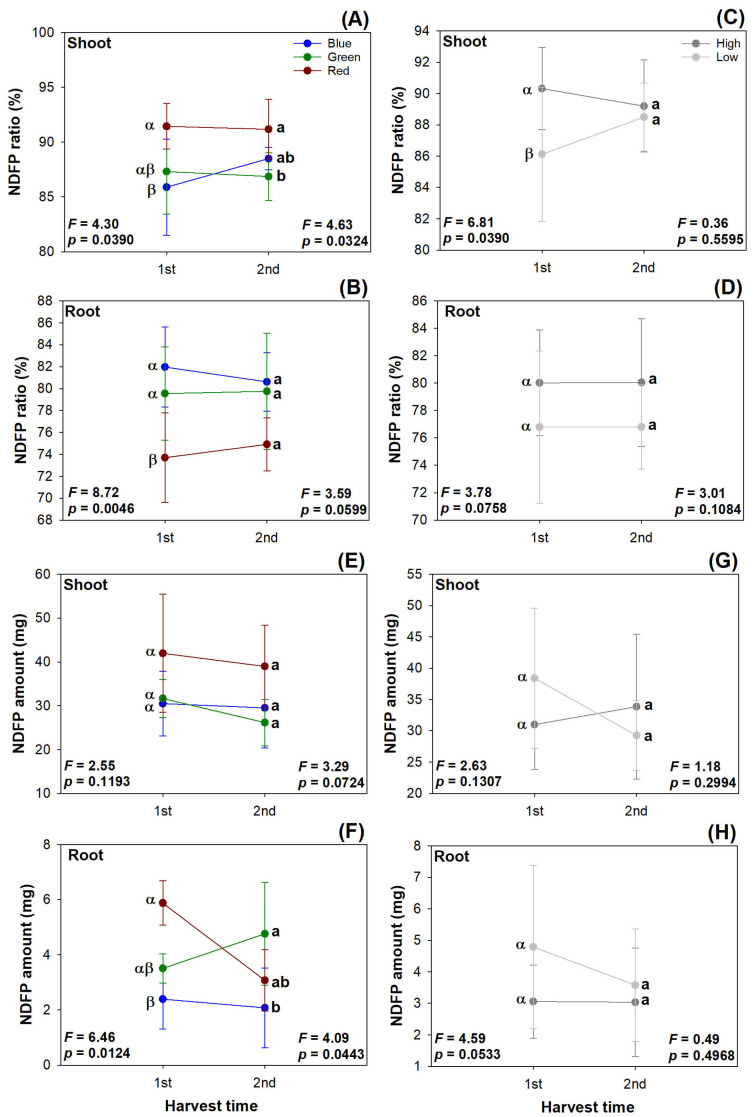
Dynamic changes in nitrogen derived from fertilizer (NDFP) ratio (**A**–**D**) and amount (**E**–**H**) in transplanted *Podocarpus macrophyllus* seedlings subjected to current light spectra (**A**,**B**,**E**,**F**) and preculture N delivery through ENL at high and low rates (**C**,**D**,**G**,**H**) in both harvests. Error bars present standard errors, above which are different letters indicating significant difference identified using Tukey test at 0.05 level. Data from first harvest are marked by different Greek letters, and those from second harvest are marked by different lowercase letters.

The red light spectrum resulted in higher shoot N being derived from the plant (NDFP) ratio compared with the blue light spectrum in the first harvest, and the red light spectrum increased the shoot NDFP ratio compared with that in the green light spectrum in the second harvest (Figure 7A). In contrast, the root NDFP ratio was lowered in the red light spectrum relative to that in the other two lighting spectra in both harvests (Figure 7B). The low rate of N delivery increased the shoot NDFP ratio compared with the high rate of N application in the first harvest, but this effect vanished in the second harvest (Figure 7C). The preculture N rate did not affect the root NDFP ratio in either of the harvests (Figure 7D).

The current light spectra did not affect the NDFP amount in either the shoot or the root parts in both harvests (Figure 7E). The root NDFP amount was higher in the red light spectrum in the first harvest, but in the second harvest, the green light spectrum changed the root NDFP amount to a higher level than that in the blue light spectrum (Figure 7F). The preculture N delivery rate did not affect the NDFP amount in either the shoot or the root parts in both harvests (Figure 7G,H).

### 2.6. Recoveries of N from Preculture Delivery and Transplant Pulse

**Figure 8:**
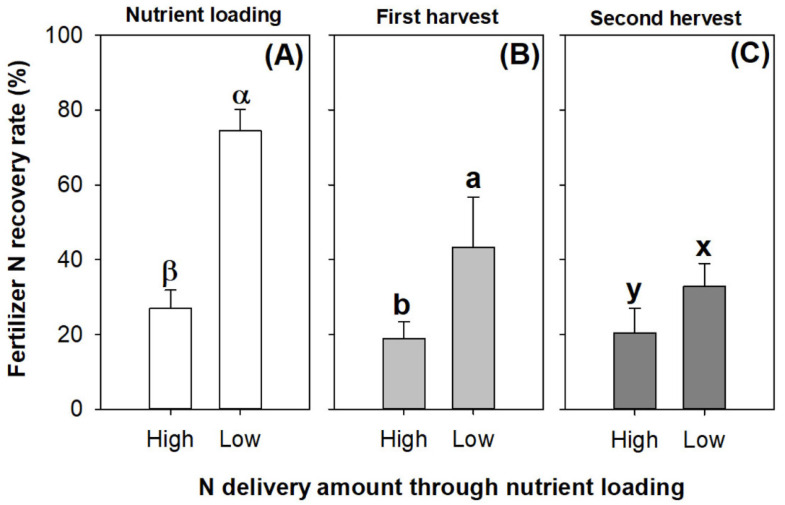
Fertilizer N recovery rates in *Podocarpus macrophyllus* seedlings subjected to high and low N application rates of preculture ENL (**A**) and post-transplantation harvests (**B**,**C**). Error bars present standard errors, above which are different letters indicating significant difference identified using Tukey test at 0.05 level. Data collected following ENL are labeled by Greek letters; data from first harvest are marked by different lowercase letters a and b; data from second harvest are marked by different lowercase letters x and y.

In the seedling culture, the low rate of N application through ENL fed the seedlings with N uptake at recovery rates from the fertilizer over 70%, while the high rate resulted in an N recovery rate of ~30% (Figure 8A). In the first harvest, the seedlings received their N delivery entirely through ENL and the N-15 pulse, which resulted in an N recovery rate from the fertilizer that was over 40% and ~20% in the low- and high-rate N application treatments, respectively (Figure 8B). In the second harvest, no new N was input to the seedlings, and the low and high N application rates resulted in N recovery rates from the fertilizer that were about ~30% and ~20%, respectively (Figure 8C). 

**Figure 9:**
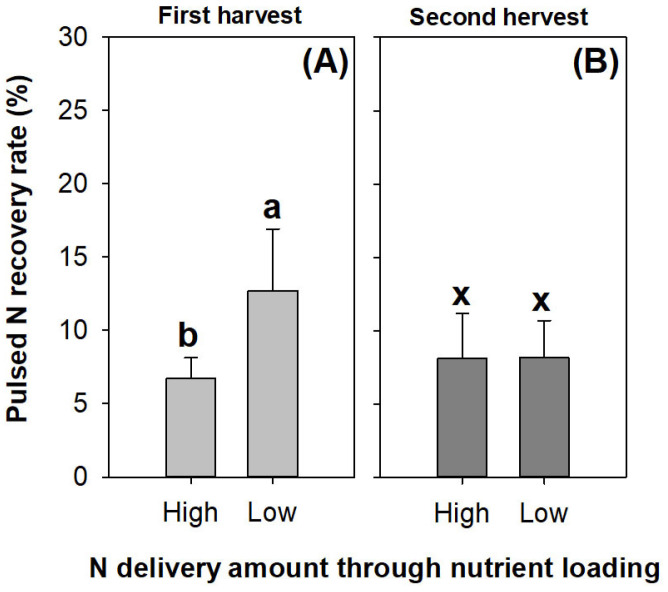
Pulsed N recovery rates in *Podocarpus macrophyllus* seedlings subjected to high and low N application rates of preculture ENL in both harvests (**A**,**B**). Error bars present standard errors, above which are different letters indicating significant difference identified using Tukey test at 0.05 level. Data from first harvest are marked by different lowercase letters a and b; data from second harvest are marked by different lowercase letters x and y.

The pulsed N was recovered at a level of ~13% in the seedlings receiving the low rate of N delivery through ENL; this was higher than that in the seedlings subjected to the high rate of N application in the first harvest (Figure 9A). However, the difference in the pulsed N recovery rate between the N application rates disappeared in the second harvest (Figure 9B).

### 2.7. Physiological Responses to Current Light Spectra and Preculture N delivery 

**Figure 10:**
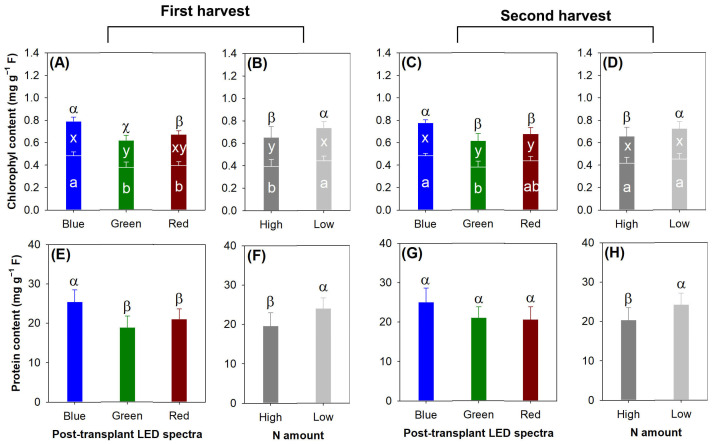
Responses of chlorophyll (a, b, a + b) content (**A**–**D**) and soluble protein content (**E**–**H**) to current light spectra (**A**,**C**,**E**,**G**) in red, green, and blue color spectra and preculture N delivery through ENL (**B**,**D**,**F**,**H**) with high and low N application rates. In cells (**A**–**D**), upper components indicate chlorophyll a content, and bottom components indicate chlorophyll b content. Error bars present standard errors. Different letters mark significant differences identified using Tukey test at 0.05 level. In cells (**A**–**D**), Greek letters mark difference in chlorophyll a + b content, lowercase letters x and y mark difference in chlorophyll a content, and lowercase letters a and b mark difference in chlorophyll b content.

In both the first and second harvests, the blue light spectrum resulted in higher chlorophyll a (first, *F* = 14.87, *p* = 0.0128; second, *F* = 8.73, *p* = 0.0046), chlorophyll b (first, *F* = 7.13, *p* = 0.0091; second, *F* = 5.32, *p* = 0.0222), and chlorophyll a + b contents (first, *F* = 20.74, *p* = 0.0001; second, *F* = 43.87, *p* < 0.0001) compared with the green light spectrum (Figure 10A,C). In addition, the chlorophyll a + b content was also elevated in the blue light spectrum relative to that in the red light spectrum in both harvests. The low-rate preculture N application resulted in higher chlorophyll a (*F* = 8.54, *p* = 0.0128), chlorophyl b (*F* = 5.41, *p* = 0.0383), and chlorophyll a + b contents (*F* = 29.25, *p* = 0.0002) compared with the high-rate N treatment in the first harvest only (Figure 10B). 

The blue light spectrum increased the protein content relative to the green and red light spectra in the first harvest (*F* = 16.35, *p* = 0.0004), but this effect vanished in the second harvest (*F* = 3.87, *p* = 0.0504) (Figure 10E,G). The low-rate preculture N application increased the protein content compared with the high-rate N treatment in both the first (*F* = 22.04, *p* = 0.0005) and second harvests (*F* = 7.42, *p* = 0.0185) (Figure 10F,H). 

**Figure 11:**
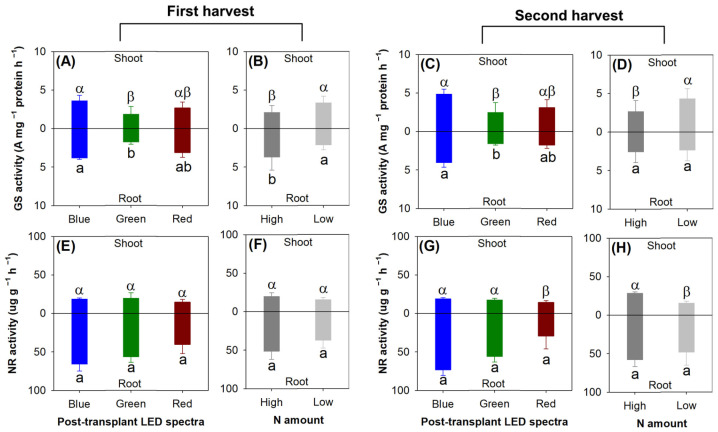
Responses of glutamine synthetase (GS) activity (**A**–**D**) and nitrate reductase (NR) activity (**E**–**H**) to current light spectra (**A**,**C**,**E**,**G**) in red, green, and blue color spectra and preculture N delivery through ENL (**B**,**D**,**F**,**H**) with high and low N application rates. Different letters mark significant differences identified using Tukey test at 0.05 level. Greek letters mark significant difference in shoot part; lowercase letters a and b mark significant difference in root part.

Among the three light spectra, the blue light spectrum increased the activity of glutamine synthetase (GS) compared with the green light spectrum in both harvests for both the shoots (first, *F* = 9.79, *p* =0.0030; second, *F* = 5.35, *p* = 0.0218) and root parts (first, *F* = 4.58, *p* =0.0332; second, *F* = 8.18, *p* = 0.0057) (Figure 11A,C). The shoot GS activity was increased in the treatments with the low rate of N delivery in both harvests (first, *F* = 13.87, *p* =0.0029; second, *F* = 7.40, *p* = 0.0186) (Figure 11B,D), but the root GS activity was decreased in the low-rate N application compared with the high-rate N treatment in the first harvest (*F* = 7.49, *p* =0.0180). 

The nitrate reductase (NR) activity was not affected by either the light spectra or the N application in the first harvest (Figure 11E,F). In the second harvest, the red light resulted in lower shoot NR activity compared with the other two spectra (*F* = 13.45, *p* = 0.0009) (Figure 11G). The low-rate N application resulted in lower shoot NR activity relative to the high-rate N treatment (*F* = 15.61, *p* = 0.0019) (Figure 11H). 

## 3. Discussion

### 3.1. Effects of ENL on N Cycling and Utilization

Element N was targeted as the core macro-nutrient in this study. It was delivered at a rate of 40 mg per seedling in the low-rate treatment and 120 mg per seedling in the high-rate treatment. The synthesized analyses of the combined biomass accumulation and reserved N state indicated that the high-rate N delivery treatment exceeded the ordinary need of N by the seedlings relative to the low-rate treatment. The parameters of the seedling growth and biomass accumulation were all higher in the low-rate treatment, which demonstrated the priority of the low-rate treatment. In this study, just two rates of N delivery through ENL were employed; consequently, it was difficult to fully identify the state of the seedling receiving the N supply. The high rate used in this study was identified as falling within an overuse range for the cultured seedlings of *Quercus rubra* [21], *Picea mariana* [22], and *P. macrophyllus* [23]. The seedlings that received an excessive N supply showed diminished features, including poor performance and a low survival rate after transplantation [7,14,18]. The overuse of N also resulted in a lowered performance in terms of growth, biomass, photosynthetic pigment, and N assimilation. These results were accompanied by higher recovery rates of fertilized N in the low-rate N application through ENL, which continued into the second harvest after transplantation. 

At the end of the culture stage, the high rate of fertilizer N recovery in the low-rate treatment was due to the higher N uptake and low amount of input. After transplantation, however, the low rate of the preculture N delivery prolonged its effect through the enhancement of the ability to not only absorb fertilizer N but also to reuse reserved N. These findings were obtained in a simulated environment that proved that the ratio of N derived from the internal reserve could be as high as 90% in the low-rate treatment. The seedlings subjected to an excessive supply reused the inner N at a low ratio of 86%. Our estimated retranslocation rates were higher than those in previous studies, which revealed ratios of 59–82% in *Pinus banksiana* seedlings [26], 73–80% in *Populus tremuloides* seedlings [25], and 23% in *Picea mariana* seedlings [27]. Nevertheless, our retranslocation rates still fell within a reasonable range because the growing substrates for the transplanted seedlings had much lower nutrient levels than those in most of the previous studies. It can be concluded that internal N retranslocation fuels the demand for new growth and that current N availability supports the magnitude of the new growth [18,28]. It is noteworthy that both the ratios in the NDFP and the recovery from pulsed N were lowered in the seedlings with toxic states in the first harvest, but the differences in these ratios all disappeared in the second harvest. These findings suggest that the effect of the preculture N input on the retranslocation continued into the first harvest when both the internal and external N supplies functioned to promote new growth. In the second harvest, the pulsed N was probably exhausted through leaching [46,47]. These results are all in agreement with the findings and viewpoint of Hawkins et al. (1999); demand for new growth drove the internal N retranslocation for reuse, and the lack of current N supply stimulated this demand, which elevated the ratio of N retranslocation in our study [28]. In the first harvest, the preculture N input still had continuous effects on the NDFF amount, while the NDFP amount was not responsive. These results demonstrate that the actual amount of N used for new growth was mainly derived from the fertilizers rather than the internal reserve. 

### 3.2. Effects of Current Light on N Cycling and Utilization

In this study, due to the current lighting spectra and preculture N delivery, rare interactive effects on the seedling parameters were found, except for height growth in the first harvest. It was revealed that the seedlings cultured in the red light spectrum with low N input through ENL had higher shoot heights than those subjected to the low-rate application plus other types of light spectra. It has been well verified that red light has a promoting effect on stem elongation in a wide range of plant species [48,49,50]. As discussed in the preceding sections, the low-rate N delivery treatment induced a less harmful effect on seedlings relative to the input of the high rate of N. The seedlings receiving the N input at a low rate grew to be healthy and responded to the red light spectrum in an ordinary manner. In the first harvest, the high rate of N delivery through ENL was shown to continue trapping seedlings in a state with an excessive N supply in all three types of spectra. During this time, the seedlings were absorbing newly pulsed N and reusing reserved N to meet the demand for new growth; the light spectra had no interactive effect; hence, the different spectra did not modify the nutritional states shaped by the preculture treatments. In the second harvest, however, the three types of spectra varied in their effects on the shaping of the nutritional states. The green light spectrum failed to increase the N content, although the N concentration was higher in the high-rate N delivery treatment; hence, the relative state was still identified as one of toxic accumulation. In the red light spectrum, the N content was increased for the following two reasons: the increase in the N concentration and the concentration increment. The blue light spectrum increased the demand for N uptake in the high-rate N delivery relative to the low-rate treatment by inducing a relative deficiency state. Overall, the red light spectrum surpassed the other two types and induced an optimum nutritional state in the second harvest. 

It is known that the current light spectra had no interactive effects on the preculture N delivery; the response of the N retranslocation resulted solely from the effect of the spectra variation. The shoots and roots showed contrasting patterns of NDFF and DNFP ratios in both harvests. The red light spectrum tended to lower the NDFF ratio in the shoots and promote it in the roots and vice versa for the NDFP ratio. It was also reported that the blue light spectrum increased the NDFP ratio relative to the red light spectrum in the shoots of the *Quercus variabilis* seedlings, and the effects were reversed in the roots [51]. This higher ratio of NDFF induced by the blue light spectrum contributed to higher levels in the total N content and concentration relative to the red light spectrum. These benefits in N uptake and allocation in the blue light spectrum all agree with the findings on *Lactuca sativa* [52]. The red light spectrum induced greater shoot biomass and shoot length relative to the other two spectra. These resulted from the high ratio of NDFP in the red light spectrum, suggesting that the higher ability to reuse internal N for new growth accounted for the shoot biomass accumulation in the red light spectrum. In the red light spectrum, the higher NDFF ratio in the roots accounted for the higher root N uptake relative to the blue light spectrum. It was also reported that root N uptake was enhanced in *Bletilla striata* seedlings by exposure to the red light spectrum [53]. Overall, the abilities to induce the NDFF ratio determined the total concentration of N in the above- and belowground parts of the *P. macrophyllus* seedlings illuminated by the blue and red light spectra, respectively. 

### 3.3. Responses of Physiological Performance

The blue light spectrum led to higher chlorophyll content compared with the other two lighting spectra, which agreed with the findings regarding shoot N concentration and GS activity. The root GS activity was increased by the exposure to the blue light spectrum rather than to the green light spectrum. All these changes agreed with the responses of the protein contents among the spectra, suggesting that the protein was mainly synthesized for the forming of GS. The findings on *Gerbera jamesonii* seedlings also demonstrated a higher chlorophyll content resulting from the acceleration of pigment synthesis [54]. Enhanced GS activity in the blue light spectrum was also reported in *Pinus koraiensis* needles [55] and *Quercus variabilis* leaves [51]. Together, these findings confirm that the blue light spectrum can enhance the assimilation of N in the form of amino acids. In the foliage organs, the blue light spectrum directly promoted N assimilation, and in the roots, the blue light spectrum induced signals that promoted N reuse from the internal reserve. These changes were confirmed not only in our study but also in a previous study conducted on *Quercus variabilis* [51]. Similarly, higher GS activities in the shoot and root parts were also found in the low-rate N delivery treatment compared with the high-rate treatment, which caused toxicity. A better ability to assimilate N in amino acids was also a positive consequence following ENL at a proper rate. 

Neither the shoots nor the roots showed varied NR activities in either of the different spectra or in the contrasting N input treatments until the second harvest. The red light spectrum induced a depression in the NR activity in needles, suggesting a lowered nitrate assimilation rate. In previous studies, it was identified that the spectra effects on NR activity depend on the length of vegetation [56,57] and that, when a significant effect occurred, it was the blue light spectrum that promoted NR activity through activation of the expression level [48]. Although the red light spectrum may exhibit night degradation of NR activity in *Gracilaria tenuistipitata*, it can also modify the circadian rhythm in the activity of NR, resulting in a decline in accumulation [58]. Moreover, in the second harvest, NR activity was higher in the seedlings with the toxic accumulation of over-supplied N. This abnormal response can be explained by two possible reasons. Firstly, the N assimilation was low in the form of amino acid in the seedlings with toxicity, leaving the major form of N assimilated as nitrate, which upregulated the NR activity [59]. This can also be a response of the physiological antagonism caused by the generation of a large amount of nitrate against the toxic state [60]. More definite conclusions require more studies to be documented with more pieces of relevant evidence.

## 4. Materials and Methods

### 4.1. Study Layout and Experimental Design

**Figure 12:**
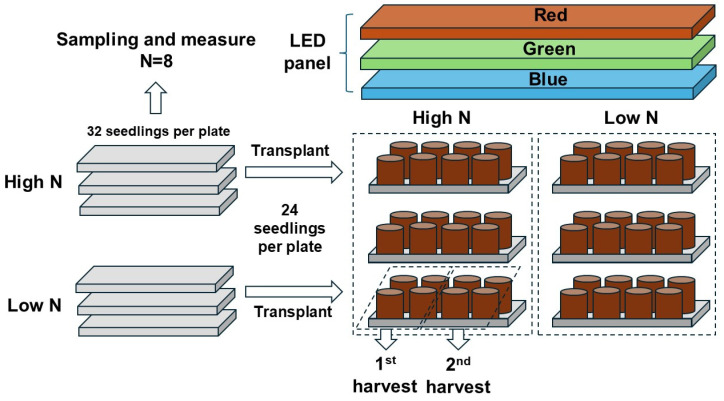
Layout of the entire process of the experiment and key information for design.

The entire process of the experimental layout and design is shown in Figure 12. The study was carried out using the random-block experimental design with two fixed factors. One factor was the rate of N delivery through ENL at the preculture stage; the other factor was the three types of light spectra simulating streetlamp light quality. A total of 32 seedlings were precultured in a planting plate, and 3 plates were arranged as 3 replicates of 1 rate measurement of the N delivery. Eight seedlings were randomly sampled at the preculture stage, leaving twenty-four seedlings for transplantation to pots. Eight pots were placed in a group exposed to one of the three light spectra, and three groups were arranged as one transplantation half-block, resulting from the twenty-four seedlings per plate during the preculture. One block comprised three groups of seedlings receiving the high-rate treatment, and the other three groups of seedlings were subjected to the low-rate treatment. Three blocks were replicated in total. Eight pots per plate were split into two random halves, with four pots in a half subjected to one of the two harvests. 

### 4.2. Plant Materials and Preculture

Seeds of *Podocarpus macrophyllus* seedlings were obtained from a forest farm of mother trees in Hangzhou (120°20′ E, 30°10′ N) [23]. The seeds were sterilized in 0.5% (*w*/*w*) potassium permanganate and maintained in distilled water for 12 h. The seeds were cleaned, dried on towels, and sown in cleaned sand at 22 °C at the moisture level of 80%. Germinant seedlings were planted in growing plugs (7 cm diameter and 13 cm height) filled with composted litter residues and perlite (3:1, *v*/*v*). The raw materials of the composts were collected from *Pinus tabuliformis* plantations distributed in eastern Liaoning [61]. Chemical analysis indicated that the properties of the growing media were characterized by a 17.93 mg/kg ammonium N content, 116.24 mg/kg nitrate N content, 4.60 g/kg total N content, 68.93 mg/kg available phosphorus (P) content, 1.14 g/kg total P content, 41.28% organic matter content, and a pH value of 6.01. Thirty-two plugs were placed on a growing plate; in total, six plates of seedlings were prepared in a greenhouse. The inner space temperature was controlled at 21–42 °C, with moisture in the range of 73–89%. When all the seedlings had grown to be over 2 cm in height, they started to receive ENL according to the following classic formula [16]:

(1) $$N_{T}=N_{I}\times (e^{rt}-1)$$

where *N_T_* is the amount of N delivered at the time *t*; *N_I_* is the initial N amount reserved within a juvenile plantlet (0.11 g seedling^−1^, adapted from Wei et al. [29]); hence, the sum of *N_T_* and *N_S_* is the total amount of N delivered to the seedlings, which were designed to be 120 and 40 mg N seedling^−1^, in treatments with contrastingly high and low rates of N delivery, respectively [17,22,23]; the coefficient *r* is the rate of the relative increment, which can be calculated as [15,47]:

(2) $$N_{t}=N_{I}\times \left(e^{rt}-1\right)-N_{t-1}$$

where *N_t_* is the amount of accumulative delivered N at the time t of the application; *N_t_*_−1_ is the accumulative amount up to the previous time. A total of 192 seedlings were cultured and received ENL. Ammonium sulfate ((NH_4_)_2_SO_4_) and potassium dihydrogen phosphate (KH_2_PO_4_) were mixed in solutions as a source of elements to achieve a balanced ratio of macro-nutrients (N-P_2_O_5_-K_2_O, 20-65-43). Micro-nutrients were added according to a previous formula [13] as follows: 1 mmol calcium chloride, 20 µmol ferric chloride, 0.6 mmol magnesium sulfate, 6 µmol manganese chloride, 0.3 µmol cupric chloride, and 0.3 µmol zinc chloride. The nitrogen supply through ENL lasted for 4 months over 16 applications at the rate of once a week; the detailed schedule is disclosed in Table 2. 

### 4.3. Seedlings, Transplant, Light Treatment, and N-15 Pulse

In September 2021, all the seedlings had received full applications of N deliveries through ENL and were acclimated to autumn conditions through the reducing of the irrigation times. Eight seedlings were randomly chosen and sampled on a planting plate, measured for height and RCD, divided into shoot and root parts, and dried at 70 °C in an oven for 72 h. The rest of the 24 seedlings per plate were transplanted to plastic pots (top inner diameter: 11 cm, height: 9.5 cm, bottom diameter: 9.5 cm), at 1 seedling per pot; each pot was filled with moist perlites. The transplanted seedlings were maintained in the greenhouse at a temperature in the range of 19 to 31 °C and a moisture level of 60 ± 11%. 

One week after transplantation, the seedlings were exposed to different lighting spectra to simulate the variation in the illuminating qualities. A light-emitting diode (LED) panel (120 cm length × 50 cm width) was used to provide artificial illumination over a group of two groups of potted seedlings (*n* = 16) (Figure 12). Diodes emitting pure red, green, and blue lights were controlled by three transformers, which controlled the electrical currents of three types of lights; their combinations created the varied light spectra. The light qualities were adapted from data that were simulated from natural conditions [62] but fell within the range of illumination that streetlamps provide [8]. Briefly, the red light spectrum was created by 20% red band, 10% green band, and 30% blue band components; the green light spectrum was created by 20% red band, 100% green band, and 10% blue band components; the blue light spectrum was created by 20% red band, 40% green band, and 20% blue band components. The photosynthetic photon flux density (PPFD) in these three types of lighting spectra ranged from 97 to 100 µmol/m^2^/s. To simulate night light illumination, the seedlings were exposed to sunlight during the daytime and subjected to LED light from 19:00 pm to 24:00 pm, following the streetlamp photoperiod [8]. 

To differentiate pulsed N from internal N for reuse by the new growth demand, ^15^N labeled ammonium-^15^N nitrate (10.10 atom%) was pulsed to the surface of the perlites in four points of a pot using solutions containing 250 ppm N (Shanghai Res. Inst. Chem. Indus. Co., Ltd., Shanghai, China). A 5 mL pipettor was used to inject pulse solutions to the surface of the perlites with no contamination of the plant tissues. An isotope of N-15 was pulsed once a day for 30 days and pulsed with water buffers for 2 days [63]. Half of the pulsed seedlings were harvested for the first time over the 30 days, and the other half were harvested 87 days later, assuming that the pulsed N had been fully depleted through leaching loss [47]. Again, the harvested seedlings were measured for height and RCD and dried for further analysis. 

### 4.4. Calculation and Parameter Determination

Vector analysis was used to estimate the relative nutritional states from the low-rate N delivery through ENL to the high-rate treatment, according to the methodology of Salifu and Timmer (2003) [27]. In this study, the ^15^N atom% (*δ*%) was calculated as [63]:

(3) δ%=N15N15+N14×100%

Therefore, the NDFF ratio (*NDFF*%) can be calculated as [27]:

(4) $$NDFF\%=\frac{{\delta \%}_{Sample}-{\delta \%}_{Standard}}{{\delta \%}_{Pulse}-{\delta \%}_{Standard}}\times 100\%$$

where *δ*%*_Sample_* is the ^15^N abundance in the sampled seedling parts; *δ*%*_Pulse_* is the ^15^N enrichment in the pulse source (10.10%); *δ*%*_Standard_* is the ambient dose at the standard value of 0.37% (~0.366%) [63]. Continuously, the NDFF amount (*A_NDFF_*) can be calculated as:

(5) $$A_{NDFF}=NDFF\%\times {DM}_{Bio}\times N_{DM}$$

where *DM_Bio_* is the dry mass biomass in the shoot or root parts of a seedling, and *N_DM_* is the N concentration in the same part. The NDFP ratio (*NDFP*%) can be calculated as:

(6) NDFP%=100%−NDFF%

Hence, the NDFP amount (*A_NDFP_*) can be calculated as:

(7) $$A_{NDFP}=NDFP\%\times {DM}_{Bio}\times N_{DM}$$

### 4.5. Chemical Analysis

The total N concentration was analyzed using the Kjeldahl method [64]. The abundance of ^15^N was analyzed using a continuous flow isotope ratio mass spectrometer (Stable Isotope Facility, UC Davis, Berkeley, CA, USA). The chlorophyll a and b contents and the soluble protein contents were determined using the methods described by Nosheen et al. (2018) [65]. The activity of the GS was assayed using a method adapted from Gao et al. (2021) [51]. The activity of the NR was assayed using Da Matta et al.’s method (1999) [66]. 

### 4.6. Statistical and Data Processing

The data were processed and analyzed using the statistics software of SAS v.9.4 (SAS Inc., Cary, NC, USA). One-way analysis of variance (one-way ANOVA) was employed to analyze the effects of ENL with contrasting rates of N delivery on the seedling parameters at the end of preculture. Mixed-model ANOVA was employed to analyze the interactive effects of the current light spectra and the preculture N treatment on the transplanted seedling parameters by splitting the data from both harvests. When a significant effect was indicated, the results were averaged and compared using the Tukey test with the critical value of significance at the level of 0.05. 

## 5. Conclusions

In this study, we conducted a simulated experiment to simulate the conditions of a preculture of *Podocarpus macrophyllus* seedlings using ENL and transplantation to city stands exposed to night illumination. The current light spectra and preculture N delivery through ENL had no interactive effects on most of the seedling parameters, except for shoot length in the transplanted seedlings. A high rate of 120 mg N seedling^−1^ induced a toxic state in the seedlings, which further resulted in reductions in growth, biomass accumulation, internal N reuse, N recovered from fertilizer, N assimilation, and photosynthetic pigments. On the other hand, the blue light spectrum tended to benefit the shoot N concentration, and the red light spectrum promoted the root N uptake, both of which resulted from the ratio of the N retained from fertilizer (15–27%). The blue light spectrum also showed that it prioritized the induction of a higher accumulation of photosynthetic pigment and amino acid assimilation relative to the other two spectra. Overall, when using ENL for the culture of *P. macrophyllus* seedlings, a rate of N delivery lower than 120 mg seedling^−1^ should be considered. Transplantation to a city stand exposed to streetlamps in a red light spectrum that can fully retranslocate internal N for new growth at a steady nutritional state is recommended. 

## Figures and Tables

**Table 1:** Summary of parameters for *Podocarpus macrophyllus* seedlings subjected to nutrient loading at contrasting N delivery rates, with difference identified using analysis of variance.

Seedling Parameters	N ^1^ Delivery Rate		ANOVA ^2^	
	High	Low	*F* Value	*p* Value
Shoot height (cm)	8.88 ± 1.25 b ^3^	11.33 ± 2.64 a	8.55	0.0065
Root-collar diameter (mm)	2.44 ± 0.26 b	2.74 ± 0.28 a	7.32	0.0111
Shoot biomass (g)	1.82 ± 0.08 b	2.54 ± 0.25 a	26.81	0.0021
Root biomass (g)	0.38 ± 0.08 a	0.48 ± 0.07 a	3.05	0.1312
Root to shoot biomass ratio	0.21 ± 0.03 a	0.19 ± 0.02 a	0.81	0.4028
Shoot N concentration (mg g^−1^ DW)	15.45 ± 1.98 a	10.38 ± 1.19 b	16.78	0.0064
Root N concentration (mg g^−1^ DW)	10.43 ± 1.42 a	7.63 ± 0.59 b	11.59	0.0144
Shoot N content (mg)	28.25 ± 4.63 a	26.17 ± 1.65 a	0.63	0.4586
Root N content (mg)	4.04 ± 1.33 a	3.64 ± 0.64 a	0.27	0.6241

^1^ N, nitrogen; ^2^ ANOVA, analysis of variance; ^3^ different letters for a seedling parameter along a row indicate significant difference according to Tukey test at 0.05 level.

**Table 2:** Schedule of chemical delivery through 16 times of applications to achieve exponential nutrient loading on *Podocarpus macrophyllus* seedlings.

Time of Application	Chemical Delivery Amount (g time^−1^ plate^−1^) ^1^			
	Low Rate		High Rate	
	(NH_4_)_2_SO_4_	KH_2_PO_4_	(NH_4_)_2_SO_4_	KH_2_PO_4_
1	0.08	0.12	0.08	0.1
2	0.1	0.14	0.1	0.13
3	0.12	0.16	0.13	0.17
4	0.14	0.18	0.17	0.22
5	0.16	0.22	0.22	0.29
6	0.2	0.26	0.29	0.39
7	0.22	0.3	0.38	0.51
8	0.26	0.36	0.5	0.66
9	0.32	0.42	0.65	0.87
10	0.36	0.5	0.86	1.14
11	0.44	0.58	1.12	1.49
12	0.52	0.68	1.47	1.96
13	0.6	0.8	1.93	2.57
14	0.7	0.94	2.53	3.37
15	0.84	1.1	3.32	4.41
16	0.98	1.3	4.35	5.78
Total (g plate^−1^)	6.04	8.06	18.1	24.07

^1^ There were 32 seedlings cultured on a planting plate.

## Data Availability

Data are contained within the article.
